# Proteasome impairment in neural cells derived from HMSN-P patient iPSCs

**DOI:** 10.1186/s13041-017-0286-y

**Published:** 2017-02-15

**Authors:** Nagahisa Murakami, Keiko Imamura, Yuishin Izumi, Naohiro Egawa, Kayoko Tsukita, Takako Enami, Takuya Yamamoto, Toshitaka Kawarai, Ryuji Kaji, Haruhisa Inoue

**Affiliations:** 1 Center for iPS Cell Research and Application (CiRA), Kyoto University, Kyoto, 606-8507 Japan; 2 Department of Clinical Neuroscience, Institute of Biomedical Sciences, Tokushima University Graduate School, Tokushima, 770-8503 Japan

**Keywords:** HMSN-P, TFG, UPS, Neurodegeneration, iPSCs, Gene correction, CRISPR-Cas9

## Abstract

Hereditary motor and sensory neuropathy with proximal dominant involvement (HMSN-P) is caused by a heterozygous mutation (P285L) in *Tropomyosin-receptor kinase Fused Gene* (*TFG*), histopathologically characterized by progressive spinal motor neuron loss with TFG cytosolic aggregates. Although the TFG protein, found as a type of fusion oncoprotein, is known to facilitate vesicle transport from endoplasmic reticulum (ER) to Golgi apparatus at ER exit site, it is unclear how mutant TFG causes motor neuron degeneration. Here we generated induced pluripotent stem cells (iPSCs) from HMSN-P patients, and differentiated the iPSCs into neural cells with spinal motor neurons (iPS-MNs). We found that HMSN-P patient iPS-MNs exhibited ubiquitin proteasome system (UPS) impairment, and HMSN-P patient iPS-MNs were vulnerable to UPS inhibitory stress. Gene correction of the mutation in *TFG* using the CRISPR-Cas9 system reverted the cellular phenotypes of HMSN-P patient iPS-MNs. Collectively, these results suggest that our cellular model with defects in cellular integrity including UPS impairments may lead to identification of pathomechanisms and a therapeutic target for HMSN-P.

**Electronic supplementary material:**

The online version of this article (doi:10.1186/s13041-017-0286-y) contains supplementary material, which is available to authorized users.

## Introduction

Hereditary motor and sensory neuropathy with proximal dominant involvement (HMSN-P) is an autosomal-dominant disease first described in patients from the Okinawa Islands of Japan [1]. The clinical manifestation of HMSN-P is characterized by late-onset progressive muscle weakness of proximal limbs with widespread fasciculations, shared with those of amyotrophic lateral sclerosis (ALS), and the patients eventually require artificial ventilation due to respiratory failure [2, 3]. The neuropathological findings of severe neuronal loss and gliosis in the spinal anterior horns and nuclei of the brainstem were reported, and as in ALS, ubiquitinated aggregates in the cytoplasm of surviving spinal motor neurons (MNs) were observed in HMSN-P [4]. A heterozygous mutation (P285L) in *Tropomyosin-receptor kinase Fused Gene* (*TFG*) was identified as the gene responsible for HMSN-P, and the pathological hallmark of HMSN-P is the formation of cytosolic inclusions of TFG protein in spinal MNs [5].

TFG forms octamers that facilitate the co-assembly of SEC 16 and COP II and work as a scaffolding protein to support vesicle trafficking from endoplasmic reticulum (ER) to Golgi apparatus at ER exit sites in various tissues including brain and spinal cord [6–8]. *TFG* mutations are related to various other neurodegenerative diseases besides HMSN-P. Homozygous *TFG* R106C mutation was identified in hereditary spastic paraplegia [9], and heterozygous *TFG* G269V mutation was found in Charcot-Marie-Tooth disease type 2 (CMT2) [10].

*TFG* P285L mutation is a heterozygous mutation located in the proline/glutamine (P/Q)-rich domain, which is essential for protein-to-protein interaction, and gains of toxic TFG functions in the region may be associated with progressive spinal MN degeneration. However, the mechanisms of how mutant TFG (mt TFG) contributes to MN degeneration in HMSN-P have not been elucidated. Recent developments in induced pluripotent stem cell (iPSC) technology [11] and the gene editing technique with the clustered regularly interspaced short palindromic repeat (CRISPR) and CRISPR associated 9 (Cas9) endonuclease systems [12] enable us to model neuronal disease and investigate the cellular phenotypes of neurons with the patient genetic background in vitro [13–15].

Here we generated iPSCs from HMSN-P patients and differentiated the iPSCs into spinal MNs. We found that HMSN-P patient spinal MNs presented ubiquitin proteasome system (UPS) impairment and cellular vulnerability. Gene correction of *TFG* P285L mutation by CRISPR-Cas9 restored these cellular phenotypes.

## Results

### Generation of HMSN-P patient iPSCs and spinal MN differentiation

**Fig. 1:**
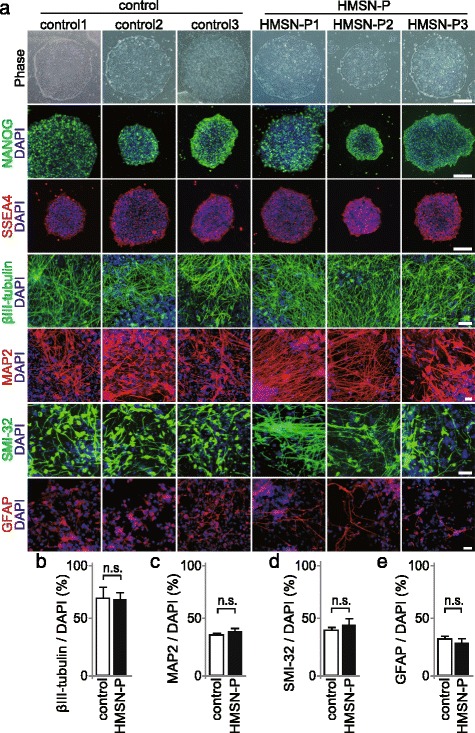
Generation of iPSCs and spinal MN differentiation. **a** iPSCs were generated from healthy control individuals and HMSN-P patients with *TFG* P285L mutation. Control and HMSN-P patient iPSCs were morphologically identical to human ESCs and expressed the pluripotent stem cell markers NANOG and SSEA4. Nucleus was stained with DAPI. Scale bars = 200 μm. Differentiated MNs were stained with neuronal marker βIII-tubulin, MAP2, and spinal MN marker SMI-32. Glial cells were stained with GFAP. Scale bars = 50 μm. **b** - **e** Proportions of control and HMSN-P patient neurons stained positive for βIII-tubulin (**b**), MAP2 (**c**), SMI-32 (**d**), and GFAP (**e**) (*n* = 3, n.s. by Student *t*-test). There were no significant differences between control and HMSN-P groups. Error bars are ± s.e.m., n.s.: not significant

We generated iPSCs from healthy control subjects and HMSN-P patients with *TFG* P285L mutation (Table 1, Additional file 1: Figure S1A). The generated iPSCs expressed pluripotent stem cell markers (Fig. 1a). The global gene expression profiles of control and HMSN-P patient iPSCs were comparable to human embryonic stem cells (ESCs) and differed from somatic cells. We analyzed mRNA expression levels of each of the iPSC clones using RNA-seq analysis, and confirmed that the significant differences were not observed among iPSCs generated from human dermal fibroblasts (HDFs) and iPSCs generated from peripheral blood mononuclear cells (PBMCs) (Additional file 1: Figure S1B), as shown previously [16]. Pluripotency of each iPSC clone was confirmed by in vitro three-germ layer assay (Additional file 1: Figure S1C).

**Table 1:** Characteristics of iPSC clones

	control1	control2	control3	HMSN-P1	HMSN-P2	HMSN-P3
Clone name at establishment	TIG107	HC2EL5	hc3NORA	ALS43EL1	ALS44E1	ALS44E9
Control or HMSN-P	control	control	control	HMSN-P	HMSN-P	
Gender	female	male	female	male	female	
Age at biopsy	81	64	65	48	52	
Disease duration	N.A.	N.A.	N.A.	5 years	12 years	
Proximal muscle weakness	N.A.	N.A.	N.A.	+	+	
Bulbar symptoms	N.A.	N.A.	N.A.	-	-	
Respiratory failure	N.A.	N.A.	N.A.	*-*	-	
Sensory disturbance	N.A.	N.A.	N.A.	+	+	
Genotype	N.A.	N.A.	N.A.	*TFG* (P285L)	*TFG* (P285L)	
Origin	HDFs	PBMCs	PBMCs	HDFs	HDFs	
Reprogramming	retrovirus	episomal	episomal	episomal	episomal	

*HMSN-P* hereditary motor and sensory neuropathy with proximal dominant involvement, *HDFs* human dermal fibroblasts, *PBMCs* peripheral blood mononuclear cells, *iPSC* induced pluripotent stem cell, *N.A.* not applicable

These iPSCs were differentiated into spinal MNs by serum-free floating culture of embryoid body-like aggregates with quick re-aggregation (SFEBq) method [17, 18]. There were no significant differences between control and HMSN-P in their differentiation capacity into spinal MNs and glial cells (Fig. 1a–e). We analyzed the cellular phenotypes of HMSN-P using these iPSC-derived neural cells with spinal MNs (iPS-MNs).

### HMSN-P patient iPS-MNs exhibited UPS impairment and vulnerability

**Fig. 2:**
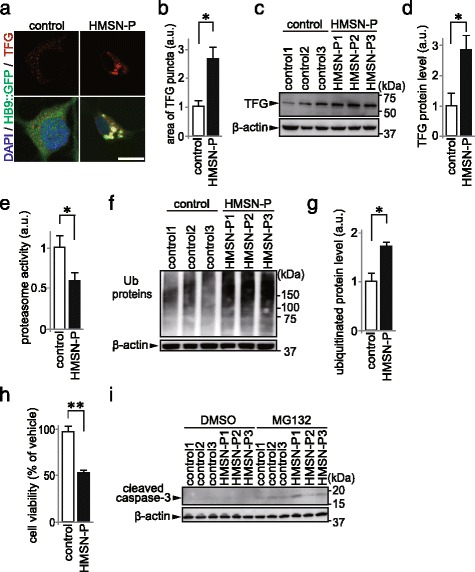
Cellular phenotypes in HMSN-P patient iPS-MNs. **a** Immunostaining for TFG in purified MNs by HB9::GFP sorting. Scale bar = 10 μm. **b** Quantification of area of TFG-positive puncta in purified MNs by HB9::GFP sorting measured by high-content analysis (*n* = 3, *p < 0.05 by Student *t-*test). Error bars are ± s.e.m. **c** Immunoblot analysis of TFG in control and HMSN-P patient iPS-MNs. β-actin was used as loading control. **d** Quantification of the TFG protein levels in control and HMSN-P patient iPS-MNs (*n* = 3, *p < 0.05 by Student *t*-test). **e** Proteasome activity of control and HMSN-P patient iPS-MNs (*n* = 3, *p < 0.05 by Student *t-*test). Error bars are ± s.e.m. **f** Immunoblot analysis of ubiquitinated proteins in control and HMSN-P patient iPS-MNs. β-actin was used as loading control. **g** Quantification of the level of HMW ubiquitinated proteins in control and HMSN-P patient iPS-MNs (*n* = 3, *p < 0.05 by Student *t-*test). Error bars are ± s.e.m. **h** Survival of control and HMSN-P patient iPS-MNs after MG132 exposure (*n* = 3, ** *p* < 0.01, by Student *t*-test). **i** Immunoblot analysis of cleaved caspase-3 in control and HMSN-P patient iPS-MNs exposed to vehicle or MG132. β-actin was used as loading control. DMSO: dimethyl sulfoxide

According to the findings in postmortem tissue from a HMSN-P patient, TFG aggregates were accumulated in spinal MNs [5], forecasting impairment of UPS [19]. We investigated whether HMSN-P patient iPS-MNs exhibited TFG aggregates. To evaluate the aggregation of TFG protein, we measured the area of TFG puncta in SMI-32-positive neurons, and dot-like TFG puncta were detected in both control and HMSN-P patient SMI-32-positive neurons. The area of TFG puncta did not differ between control and HMSN-P (Additional file 2: Figure S2A, 2B), although there was a trend toward a larger area of TFG puncta in HMSN-P patient SMI-32-positive neurons. In some neurodegenerative disease models using iPSCs, it is required to provide cellular stress so as to recapitulate disease phenotypes such as accumulation of pathological proteins and cell death [20]. As a cellular stress, to remove trophic supports from surrounding cells including glial cells to spinal MNs, we purified spinal MNs labeled with lentivirus vector expressing green fluorescent protein (GFP) under control of HB9 promoter (HB9::GFP) [21], and succeeded in finding TFG aggregates in spinal MNs. The area of TFG aggregates increased significantly in HMSN-P-patient purified MNs compared to controls (Fig. 2a, b), although TFG aggregates in HMSN-P patient purified MNs were not increased significantly when purified MNs were co-cultured with glial cells (Additional file 2: Figure S2C, 2D). These TFG aggregates were not merged with ubiquitin (Additional file 2: Figure S2E). Next, we evaluated the protein levels of TFG without additional cellular stress. Immunoblot analysis of iPS-MNs showed the TFG protein level to be significantly increased in HMSN-P compared to controls (Fig. 2c, d). These results demonstrated that TFG protein levels were increased in HMSN-P, and that additional cellular stress accelerated TFG aggregations.

Accumulation of aberrant protein is caused by dysfunction of protein degradation systems such as UPS [22]. We measured UPS activity of iPS-MNs, and found that UPS activity was impaired in HMSN-P compared to control (Fig. 2e). Ubiquitinated proteins were increased in HMSN-P, showing accumulation of high molecular weight (HMW) proteins as detected by anti-ubiquitin antibody (Fig. 2f, g). These results suggest that HMSN-P patient iPS-MNs exhibited UPS dysfunction.

To investigate the association of UPS impairment with spinal MN vulnerability, we evaluated spinal MN survival. In a culture duration of as long as 3 months, we could not detect any vulnerability of HMSN-P patient iPS-MNs compared to control. Clinically, the onset of HMSN-P is after middle age [1]. It may be needed cellular stress as shown in aging process to detect vulnerability of HMSN-P patient iPS-MNs. It is reported that UPS impairment progresses according to aging [23]. Therefore, we added proteasome inhibitor MG132 as a stress inducer to enhance UPS impairment in order to examine whether HMSN-P patient iPS-MNs presented neuronal vulnerability. The number of surviving HMSN-P patient iPS-MNs was significantly decreased by MG132 exposure compared to controls (Fig. 2h). Immunoblot analysis showed that the level of cleaved caspase-3 was increased in HMSN-P patient iPS-MNs after MG132 exposure (Fig. 2i). We also confirmed the vulnerability using another specific proteasome inhibitor, bortezomib (Additional file 3: Figure S3). These data suggest that HMSN-P patient iPS-MNs are vulnerable under UPS inhibitory stress in comparison to control.

### Correction of *TFG* P285L mutation ameliorated cellular phenotypes of HMSN-P patient iPS-MNs

**Fig. 3:**
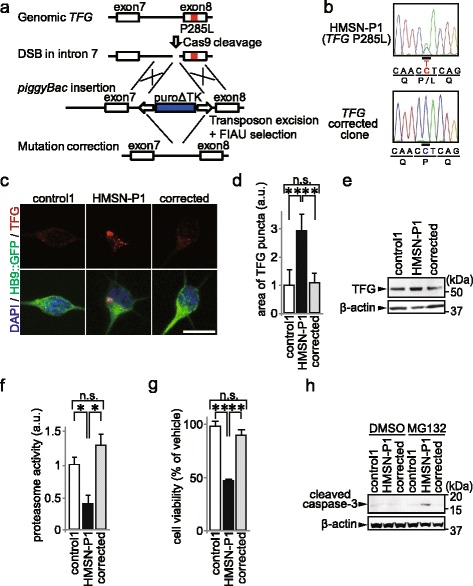
Gene correction of *TFG* P285L mutation and improvement of cellular phenotypes in HMSN-P. **a** Gene targeting strategy used to generate mutation-corrected iPSC clone. CRISPR-Cas9 targeting the *TFG* locus created a double strand break upstream of exon 8. Homologous recombination of the genomic locus with a targeting plasmid with control sequence of exon 8 coupled puroΔTK replaced the *TFG* P285L mutant allele. After puromycin selection, the resistance cassette was removed by transposon expression and FIAU selection. DSB: double strand break, FIAU: 1-(2-Deoxy-2-fluoro-β-D-arabinofuranosyl)-5-iodouracil. **b** Sequencing of exon 8 of *TFG* in mutation-corrected iPSCs showed the correction of the *TFG* P285L mutation. **c** Immunostaining for TFG in purified MNs by HB9::GFP sorting. Scale bar = 10 μm. **d** Quantification of area of TFG-positive puncta in purified MNs by HB9::GFP sorting measured by high-content analysis (*n* = 9, **p < 0.01, one-way ANOVA followed by Tukey’s multiple comparison *post hoc* test). Error bars are ± s.e.m. **e** Immunoblot analysis of TFG in control, HMSN-P patient, and mutation-corrected iPS-MNs. β-actin was used as loading control. **f** Proteasome activity in control, HMSN-P patient, and mutation-corrected iPS-MNs (*n* = 3, * *p* < 0.05, one-way ANOVA followed by Tukey’s multiple comparison *post hoc* test). Error bars are ± s.e.m. **g** Survival of control, HMSN-P patient, and mutation-corrected iPS-MNs after MG132 exposure (*n* = 3, **p < 0.01, one-way ANOVA followed by Tukey’s multiple comparison *post hoc* test). Error bars are ± s.e.m. **h** Immunoblot analysis of cleaved caspase-3 in control, HMSN-P patient, and mutation-corrected iPS-MNs after exposure of vehicle or MG132. DMSO: dimethyl sulfoxide

To determine whether the phenotypes we observed in HMSN-P patient iPS-MNs were dependent on the *TFG* P285L mutant allele, we applied CRISPR-Cas9 mediated gene targeting strategy to correct the mutation in a HMSN-P patient iPSC clone, HMSN-P1. The process of targeted gene correction is illustrated in Fig. 3a. We seamlessly generated *TFG* P285L mutation-corrected HMSN-P patient iPSCs (corrected) according to previous reports [24]. Targeted gene correction was confirmed by restoration of the original intron 7 and exon 8 without any exogenous sequence by DNA sequencing (Fig. 3b).

We differentiated mutation-corrected iPSCs into spinal MNs, and found that the area of TFG aggregates in the mutation-corrected purified MNs decreased significantly compared to HMSN-P patient purified MNs (Fig. 3c, d). The TFG protein levels were decreased by the gene correction detected by immunoblot analysis (Fig. 3e). UPS activity in HMSN-P was significantly recovered by the gene correction (Fig. 3f). Survival of iPS-MNs after MG132 exposure was significantly increased (Fig. 3g), and the level of cleaved caspase-3 was decreased (Fig. 3h) by the gene correction. These data proved that *TFG* P285L mutation caused the HMSN-P cellular phenotypes associated with UPS impairment.

## Discussion

We presented cellular phenotypes associated with the UPS impairment of HMSN-P patient iPS-MNs, and showed that gene correction of *TFG* P285L mutation ameliorated the mt TFG-associated cellular phenotypes.

UPS is a major pathway for the protein degradation system to handle neurodegeneration-related proteins [22]. UPS dysfunction in 26S proteasome-mediated protein clearance [25] and aberrant accumulation of ubiquitinated proteins in neurodegenerative diseases including prion disease, Alzheimer’s disease, and ALS are reported [26–28]. In prion disease, prion protein binds directly to 20S core components in the proteasome, interferes with gate opening for substrate entry that leads to defects of peptide hydrolysis, and impairs proteasomal degradation of ubiquitinated proteins [26]. Tau protein, accumulated in Alzheimer’s disease brain, also directly binds to 20S core components and inhibits UPS activity [27]. TDP-43, a MN disease-relevant protein, impairs UPS activity, although details of the mechanism for this are unknown [28]. Our findings of UPS impairment in HMSN-P are consistent with other neurodegenerative diseases. A mt TFG-overexpressing cellular model showing impairment of UPS [29] supports our findings. However, it is unclear how mt TFG causes UPS impairment. We speculate that mt TFG may possess direct or indirect inhibitory effects on UPS as well as other neurodegenerative diseases [19, 30]. On the other hand, we revealed that UPS impairment was observed without TFG aggregates in our experiments. TFG aggregates may not be essential for UPS impairment-associated pathomechanisms. Further investigations for the elucidation of the mechanisms of how mt TFG contributes to the impairment of UPS activity are needed.

Our iPSC models have the advantage of obtaining data from living human neurons without gene overexpression. We proved the decrease of UPS activity in HMSN-P patient neural cells generated from iPSCs directly. These findings were supported by a previous report using cancer cell lines overexpressing mt TFG indicated UPS dysfunction. Our data are important for providing evidence using human patient neural cells. Furthermore, since we should carefully consider and minimize clonal variations for disease modeling with iPSCs [31–33], we generated mutation-corrected isogenic control iPSCs, and used not only healthy control iPSCs but also isogenic control iPSCs for disease modeling, and confirmed that the observed cellular phenotypes were caused by the *TFG* mutation.

In conclusion, we found UPS impairment as the distinctive neural phenotypes of HMSN-P using patient iPSCs. Our findings suggest that this cellular model may contribute to elucidation of the disease pathomechanisms and identification of a therapeutic candidate for HMSN-P.

## Materials and methods

### Generation of iPSCs

HDFs or PBMCs from healthy control subjects and HMSN-P patients were reprogrammed by introducing the episomal vectors with OCT3/4, SOX2, KLF4, L-MYC, LIN28, and p53 carboxy-terminal dominant-negative fragment as previously described [34]. Established iPSCs were grown under feeder-free conditions on laminin-511 E8 (Nippi, Tokyo, Japan) -coated plates with StemFit AK03N (Ajinomoto, Tokyo, Japan) [35]. Another human iPSC clone, TIG107 [17], was added to the control iPSC group.

### Genotyping of *TFG*

Genotyping of *TFG* single nucleotide mutation was performed by PCR amplification of genomic DNA and directly sequenced (3500xL Genetic Analyzer, Applied Biosystems, Waltham, MA).

### RNA-seq analysis

Total RNA was extracted by RNeasy Mini Kit (Qiagen, Venlo, Netherlands), and strand-specific cDNA libraries were prepared using the TruSeq Stranded mRNA kit on a NeoPrep system (Illumina, San Diego, CA) according to the manufacturer’s instructions. The resulting libraries were sequenced (75 bp single-end) on a NextSeq 500 system (Illumina). The sequenced reads were mapped to the human reference genome (hg38) using tophat-2.1.0 with the aligner Bowtie2-2.2.5 [36, 37] after trimming adaptor sequences and low-quality bases by cutadapt-1.8.1 [38]. The number of reads mapped to each gene was counted by HTSeq-0.6.1 software [39], and then normalized with the DESeq2 R/Bioconductor package (v. 1.12.3) [40]. Hierarchical clustering analysis was performed using R software version 3.3.1.

### In vitro three-germ layer assay

iPSCs were dissociated with a cell scraper and used for embryoid body (EB) formation. Clumps of cells were transferred to suspension plates in Dulbecco’s modified Eagle’s medium/Ham’s F12 (DMEM/F12, Sigma-Aldrich, St.Louis, MO) containing 20% knockout serum replacement (KSR, Life Technologies, Waltham, MA), 2 mM L-glutamine, 0.1 mM nonessential amino acids (NEAA, Invitrogen, Waltham, MA), 0.1 mM 2-mercaptoethanol (2-ME, Life Technologies), and 0.5% penicillin and streptomycin. The medium was changed every other day. On Day 8, EBs were plated onto gelatin-coated plates and allowed to differentiate for an additional 8 days.

### Spinal MN differentiation from iPSCs

Induction of spinal MN differentiation from iPSCs was performed as previously described [17, 18]. Briefly, iPSCs were dissociated to single cells and quickly re-aggregated in U-bottom 96-well plates for suspension culture (Greiner Bio-One, Frickenhausen, Germany), pre-coated with 2% Pluronic (Sigma-Aldrich) in 100% ethanol. Aggregated EBs were cultured in 5% DFK medium [DMEM/F12 (Sigma-Aldrich), 5% KSR (Gibco, Waltham, MA), 0.1 mM NEAA (Invitrogen), 2 mM L-glutamine (Sigma-Aldrich), 0.1 mM 2-ME (Invitrogen)] with 2 μM dorsomorphin, 10 μM Y27632 (Wako Chemicals, Osaka, Japan), 10 μM SB431542 (Wako Chemicals), 3 μM CHIR99021 (Axon Medchem, Groningen, Netherlands), and 12.5 ng/ml bFGF (Wako Chemicals) for 4 days. On Day 4, 100 nM retinoic acid (Sigma-Aldrich) and 0.5 μM Smoothened ligand (Enzo Life Science, Farmingdale, NY) were added to the 5% DFK medium. On Day 11, EBs were cultured in neurobasal medium (Life Technologies) supplemented with 2% B27 without vitamin A (Life Technologies), and 100 nM retinoic acid, 0.5 μM Smoothened ligand, and 10 μM DAPT (Sigma-Aldrich) were added to a suspension culture plate. They were separated from the dish by Accutase (Nacalai Tesque, Kyoto, Japan), dissociated into a small clump or single cells, and plated at 5.0 x 10^5^cells per well onto Matrigel (Corning, Tewksbury, MA) -coated 24-well plates on Day 16, and then cultured for 2 weeks.

### Immunocytochemistry

Plated cells were fixed in 4% paraformaldehyde in phosphate-buffered saline (PBS) for 30 min. Cells were washed three times with PBS after each step and then blocked with 5% bovine serum albumin (Nacalai Tesque) for at least 1 h at room temperature. The cells were permeabilized prior to primary antibody incubation. Cells were then incubated with primary antibodies at 4 °C overnight and washed three times with PBS. The following primary antibodies were used in this assay: NANOG (1:500; ReproCELL, Yokohama, Japan), SSEA4 (1:1,000; Chemicon, Darmstadt, Germany), βIII-tubulin (1:2,000; Covance, Princeton, NJ), MAP2 (1:1,000; Millipore, Billerica, MA), SMI-32 (1:2,000; Covance), GFAP (1:1,000; DAKO, Glostrup, Denmark), TFG (1:1,000; Protein Tech, Rosemont, IL), Ubiquitin (1:1,000; DAKO), FK2 (1:1,000; MBL, Nagoya, Japan), SOX17 (1:1,000; R&D Systems, Minneapolis, MN), and αSMA (1:500; DAKO). Suitable secondary antibodies (Invitrogen) were incubated with samples at room temperature for 1 h, and washed three times with PBS. Nuclear staining was also performed with 0.5 g/mL 4′,6-Diamidino-2-Phenylindole (DAPI) for 5 min, and then the cells were washed three times with PBS. Coverslips were mounted in ProLong Gold antifade reagent (Invitrogen). Images were collected using LSM710 (Carl Zeiss, Oberkochen, Germany) or IN Cell Analyzer 6000 (GE Healthcare).

### Immunoblot analysis

iPS-MNs of each clone were harvested on Day 35 and lysed in RIPA buffer [50 mM Tris-HCl buffer, pH 8.0, 0.15 M NaCl, 1% Nonidet P-40 (NP-40), 0.5% sodium deoxycholate, 0.1% SDS] with protease inhibitor cocktail (Roche, Basel, Switzerland) and phosphatase inhibitor (Roche) on ice for 30 min. After sonication with Bioruptor (M2 mode, ON: 20 s, OFF: 20 s, 20 times), samples were centrifuged at 20,000 g for 15 min at 4 °C. The supernatant was used as sample. Each 10- or 15-μg sample was subjected to SDS-PAGE (10-20% polyacrylamide gels, BIO CRAFT, Tokyo, Japan), and separated proteins were transferred to PVDF. The membranes were incubated with primary antibodies, followed by appropriate secondary antibodies, and then visualized using ECL Prime (GE Healthcare, Chicago, IL). The images were acquired on LAS 4000 (GE Healthcare). The following primary antibodies were used in this assay: TFG (1:1,000; Protein Tech), Ubiquitin (1:1,000; DAKO), cleaved caspase-3 (1:1,000; Cell Signaling Technology, Danvers, MA), and β-actin (1:5,000; Sigma-Aldrich).

For detection of cleaved caspase-3, iPS-MNs of each clone were exposed to vehicle or 1 μM MG132 on Day 35 for 24 h. Cells were lysed in RIPA buffer and used for immunoblot analysis with anti-cleaved caspase-3 antibody.

### Purification of MNs using flow cytometry

Production, concentration and infection of HB9::GFP lentivirus vector were previously reported [17]. All sorting procedures were carried out using FACS Aria II (BD Biosciences, Franklin Lakes, NJ). After infection with HB9::GFP lentivirus on Day 20, the differentiated cells were dissociated into single cells by Accutase and resuspended in PBS with 2% FBS and 20 mM D-glucose on Day 35 for cell sorting. Dead cells were identified by DAPI staining and excluded from target cells. After gating optimized for excluding cell doublet HB9::GFP positive cells and other negative cells, targeted cells were sorted and plated onto Matrigel-coated 96-well plates at 20,000–30,000 cells/well. Sorted cells were also plated on a confluent monolayer of primary cortical mouse glia. Primary glias from P0–P1 mouse pups were prepared as described previously [41].

### Quantitating the area of TFG aggregates in cytoplasm

HB9::GFP-positive MNs or SMI-32-positive neurons were stained with anti-TFG antibody. The area of TFG puncta in cytoplasm was quantified by IN Cell Analyzer 6000 (GE Healthcare) and IN CELL Developer toolbox software 1.92 (GE Healthcare) in randomized 9- or 16-field images in each well.

### Cell survival assay

iPS-MNs of each clone on Day 35 were seeded at 3.0 x 10^4^cells per well on Matrigel-coated 96-well plates for 48 h after treatment with DMSO or 10 μM MG132, or 10 nM bortezomib (Selleck Chemicals, Houston, TX). The nucleus was stained with DAPI. Each clone was analyzed using IN Cell Analyzer 6000 (GE Healthcare) and IN CELL Developer toolbox software 1.92 (GE Healthcare) in randomized 9-field images in each well. Cell counts treated with MG132 or bortezomib were normalized to cell counts treated with DMSO in each well.

### Analysis of proteasome activity

iPS-MNs of each clone on Day 35 were seeded at 5.0 x 10^5^cells per well on Matrigel-coated 24-well plates. Whole cell lysates of iPS-MNs were lysed in 0.5% NP-40. After sonication with Bioruptor (M2 mode, ON: 20 s, OFF: 20 s, 20 times), cell lysates were centrifuged at 20,000 g for 15 min at 4 °C. The supernatant was used as sample. Proteasome activity was measured with a Proteasome Activity Assay Kit (Abcam, Cambridge, UK) following the manufacturer’s instructions.

### Generation of a mutation-corrected clone

For correcting *TFG* P285L mutation by CRISPR-Cas9, we designed a guide RNA to target the 5′-CCTAACAGTAAGACTAATT-3′ site using CRISPR Design (http://crispr.mit.edu/). The guide RNA oligonucleotide was inserted into the BamHI-EcoRI site in pHL-H1-ccdB plasmids in order to express from human H1 polymerase III promoter [12]. We inserted 5′ and 3′ homology arms with normal *TFG* gene sequence, and puromycin-resistant cassette flanked by *piggyBac* terminal repeats into pBluescript SK (+) for constructing the donor plasmid. Their nuclease activity was validated in 293 T cells by T7E1 method [42]. Target iPSCs (HMSN-P1) were pretreated with 10 μM Y-27632 (Wako Chemicals) for 1 h before electroporation. The cells were washed with PBS and treated with 0.5x TrypLE Select (Gibco) to dissociate into single cells for 4 min at 37 °C and were then neutralized with culture medium. Next, we electroporated 10 μg of pHL-H1 guide RNA expression plasmids, 10 μg of pHL-EF1α-hcSpCas9 plasmids, and 10 μg of donor plasmids into 1 x 10^6^ cells using a NEPA 21 electroporator (Nepagene, Chiba, Japan). Four days after transfection, puromycin selection was applied for 10 days. Surviving colonies were expanded and PCR was used to confirm proper cassette insertion by PCR using primers A, B, C, and D (shown in Additional file 4: Table S1). Amplified PCR bands were further analyzed by Sanger sequencing to confirm the absence of sequence alterations at the homology arms. To remove the puromycin cassette, 1 x 10^6^ cells were electroporated with 10 μg of *piggyBac* transposase expressing vector pHL-EF1α-hcPBase [43]. At 2 days after electroporation, these cells were passaged. Two days after passage, colonies were dissociated into single cells and plated at 200–1000 cells per 100-mm dish. On the following day, 1-(2-Deoxy-2-fluoro-β-D-arabinofuranosyl)-5-iodouracil (FIAU) selection was performed. Surviving colonies were expanded and PCR screening was conducted using primers A, C, and D (Additional file 4: Table S1) for proper removal of the puromycin cassette. Direct sequencing (3500xL Genetic Analyzer, Applied Biosystems) confirmed that the single nucleotide mutation was corrected.

### Statistical analysis

All data are shown as mean ± s.e.m. Comparison of two groups was analyzed using unpaired two-tailed Student’s *t*-test or paired *t*-test. One-way ANOVA was performed for each comparison, followed by Tukey’s *post hoc* tests for evaluation of pairwise group differences. A *p*-value < 0.05 was considered statistically significant. Analyses were performed with IBM SPSS statistics software (IBM, Armonk, NY); *p < 0.05, **p < 0.01.

## Additional files

Additional file 1: Figure S1. Global gene expression in iPSC clones and in vitro three-germ layer assay. (A) HMSN-P patient iPSC clones carried a *TFG* P285L mutation. (B) Comparison of global gene expression profiles of human iPSCs. Heatmap analysis and hierarchical clustering showed that the global gene expression profiles of control and HMSN-P patient iPSCs were identical to human embryonic stem cells (H9 ESCs) and different from HDFs or PBMCs. There are no significant changes in RNA expression between iPSCs generated from HDFs and PBMCs or those generated by episomal vectors or retrovirus vectors. HDFs: human dermal fibroblasts, PBMCs: peripheral blood mononuclear cells. (C) In vitro three-germ layer assay is shown. Scale bars = 100 μm. Pluripotency of each iPSC clone was confirmed by in vitro three-germ layer assay. TIG107 was reported previously [11]. (PDF 1177 kb)
Additional file 2: Figure S2. Evaluation of TFG aggregates in HMSN-P patient spinal MNs. (A) Immunostaining for TFG in control and HMSN-P patient SMI-32-positive neurons. Scale bar = 10 μm. (B) Quantification of area of TFG-positive puncta in SMI-32-positive neurons measured by high-content analysis (*n* = 3, n.s. by Student *t-*test). Error bars are ± s.e.m. (C) Immunostaining for TFG in purified MNs by HB9::GFP sorting plated on monolayer of primary cortical mouse glia. Scale bar = 10 μm. (D) Quantification of area of TFG-positive puncta in purified MNs measured by high-content analysis (*n* = 3, n.s. by Student *t-*test). Error bars are ± s.e.m. TFG aggregates were not significantly increased in purified MNs when they were co-cultured with mouse glia. (E) Immunocytochemical analysis with anti-multi-ubiquitin chain antibody (FK2) using control and HMSN-P patient iPS-MNs. Aggregations of multi-ubiquitin were not detected in HMSN-P patient iPS-MNs. (PDF 509 kb)
Additional file 3: Figure S3. Vulnerability of HMSN-P patient iPS-MNs against UPS inhibitory stress. Survival of control and HMSN-P patient iPS-MNs after bortezomib exposure (*n* = 3, ***p* < 0.01, by Student *t*-test). (PDF 222 kb)
Additional file 4: Table S1. Primers used for editing TFG gene. (DOCX 12 kb)

## Abbreviations

2-ME: 2-mercaptoethanol
ALS: Amyotrophic lateral sclerosis
Cas9: CRISPR associated 9
CRISPR: Clustered regularly interspaced short palindromic repeat
DAPI: 4′,6-Diamidino-2-Phenylindole
DMSO: Dimethyl sulfoxide
DSB: Double strand break
EB: Embryoid body
ER: Endoplasmic reticulum
ESCs: Embryonic stem cells
FIAU: 1-(2-Deoxy-2-fluoro-β-D-arabinofuranosyl)-5-iodouracil
GFP: Green fluorescent protein
HDFs: Human dermal fibroblasts
HMSN-P: Hereditary motor and sensory neuropathy with proximal dominant involvement
HMW: High molecular weight
iPSCs: Induced pluripotent stem cells
iPS-MNs: Induced pluripotent stem cells-derived neural cells with spinal motor neurons
MNs: Motor neurons
mt: Mutant
n.s.: Not significant
P/Q-rich domain: Proline/glutamine-rich domain
PBMC: Peripheral blood mononuclear cells
PBS: Phosphate-buffered saline
TDP-43: TAR DNA-binding protein 43 kDa
TFG: Tropomyosin-receptor kinase Fused Gene
UPS: Ubiquitin proteasome system
