# Supplementary figures and images for: Proteasome impairment in neural cells derived from HMSN-P patient iPSCs
Source: Mol Brain. 2017 Feb 15;10:7. doi: 10.1186/s13041-017-0286-y (PMC5310050; doi:10.1186/s13041-017-0286-y)

Figure S1

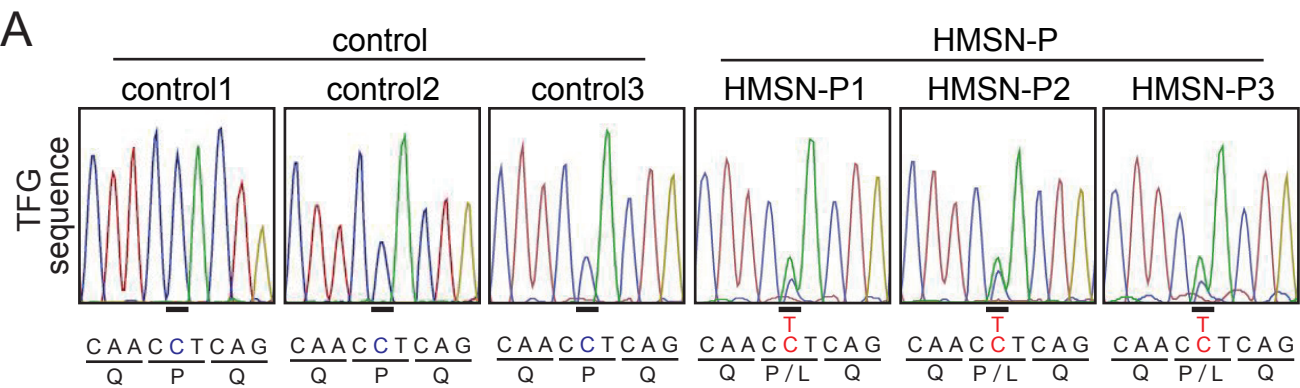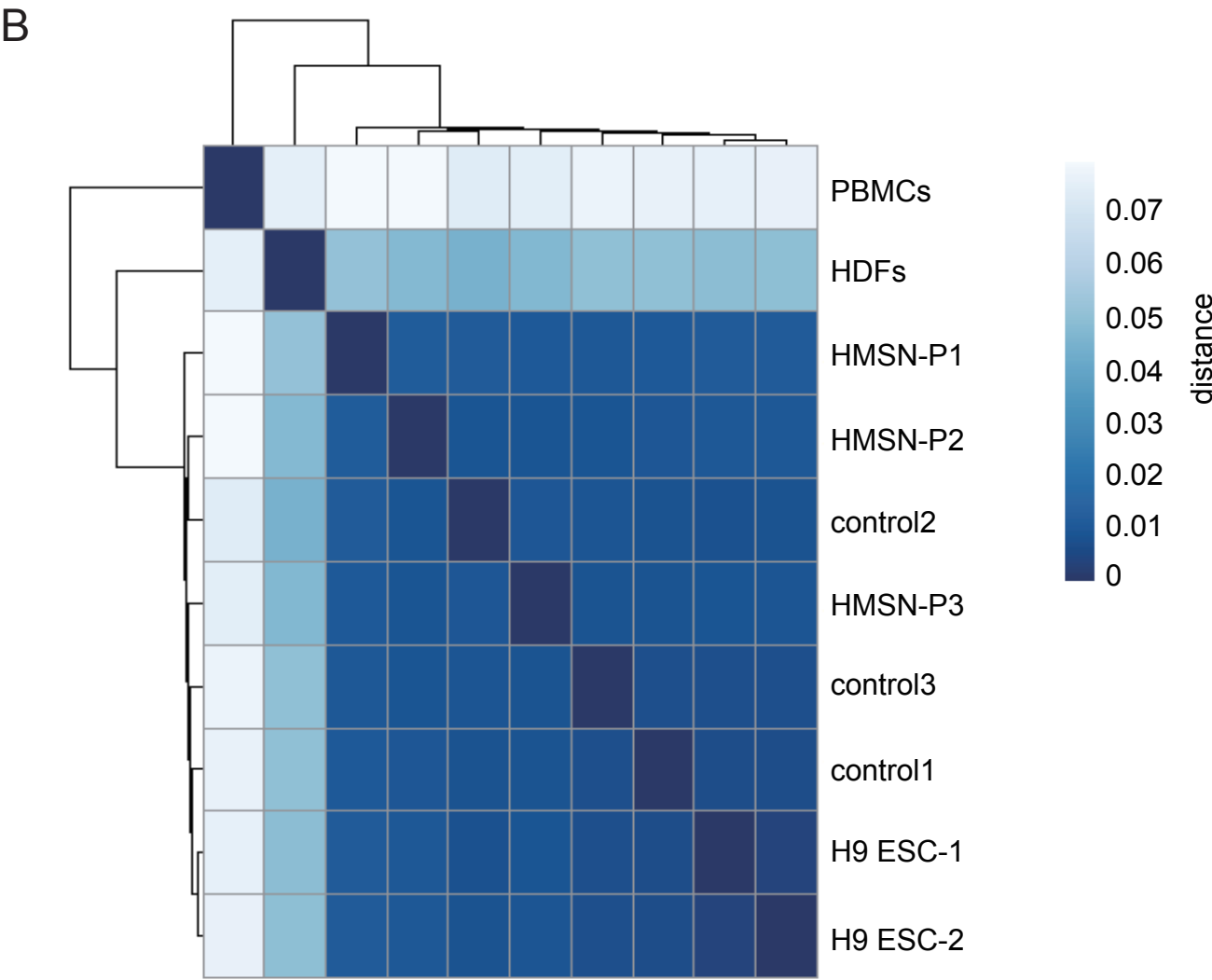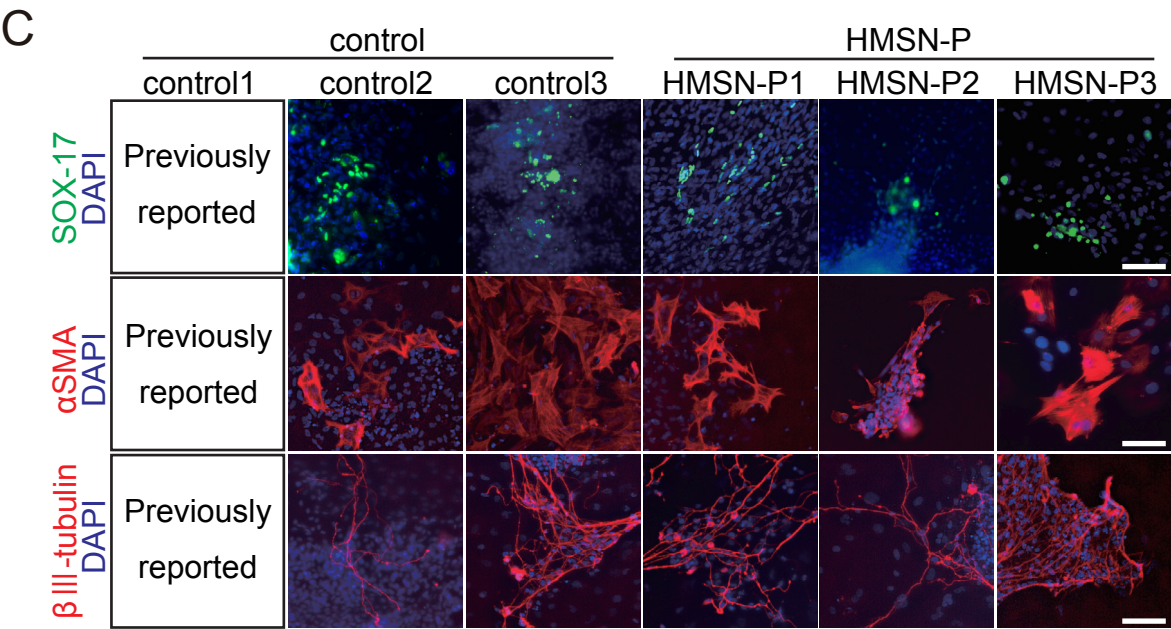

Supplement: Additional file 1: Figure S1. — Global gene expression in iPSC clones and in vitro three-germ layer assay. (A) HMSN-P patient iPSC clones carried a TFG P285L mutation. (B) Comparison of global gene expression profiles of human iPSCs. Heatmap analysis and hierarchical clustering showed that the global gene expression profiles of control and HMSN-P patient iPSCs were identical to human embryonic stem cells (H9 ESCs) and different from HDFs or PBMCs. There are no significant changes in RNA expression between iPSCs generated from HDFs and PBMCs or those generated by episomal vectors or retrovirus vectors. HDFs: human dermal fibroblasts, PBMCs: peripheral blood mononuclear cells. (C) In vitro three-germ layer assay is shown. Scale bars = 100 μm. Pluripotency of each iPSC clone was confirmed by in vitro three-germ layer assay. TIG107 was reported previously [11]. (PDF 1177 kb) [file 13041_2017_286_MOESM1_ESM.pdf]

Figure S2

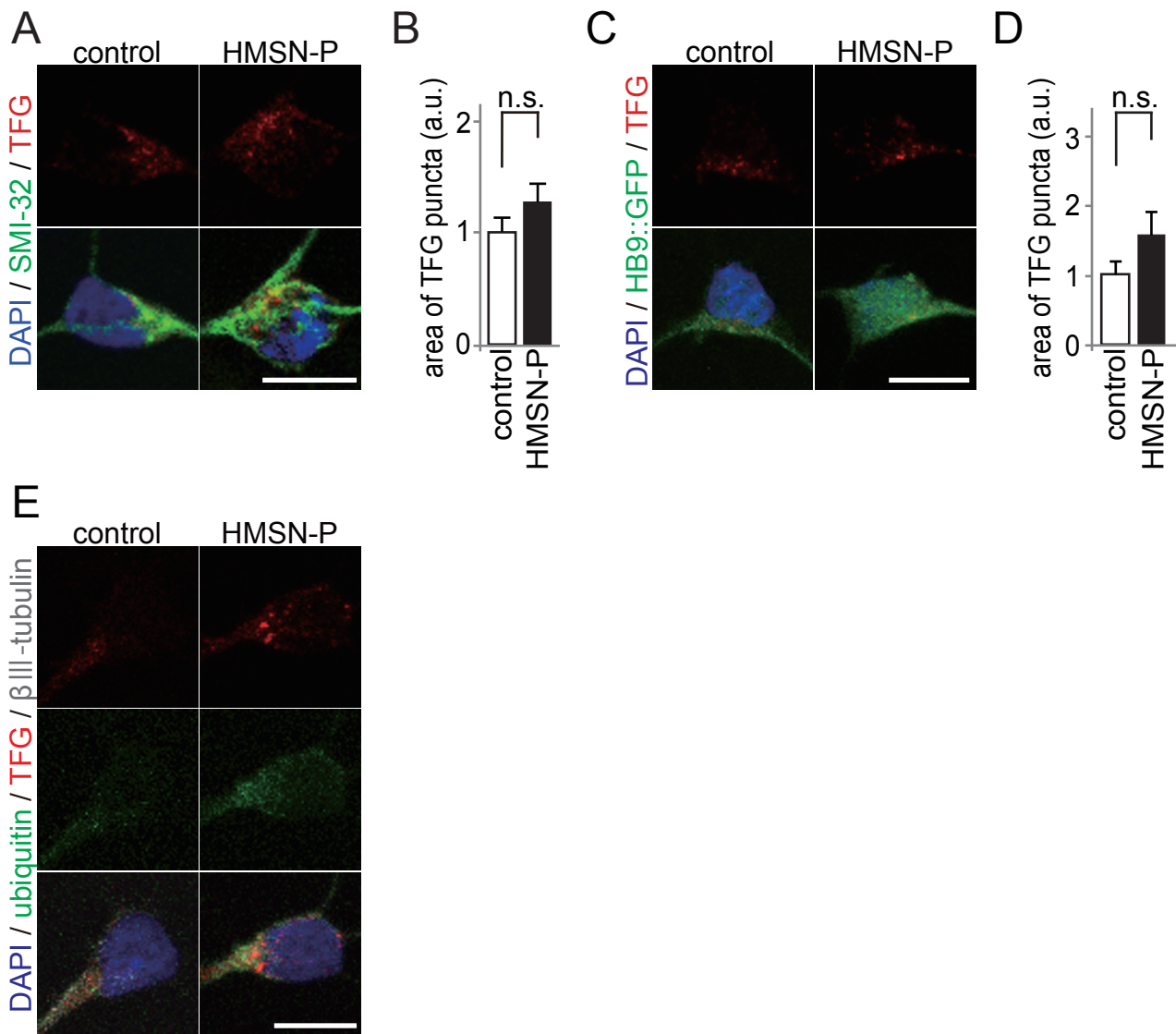

Supplement: Additional file 2: Figure S2. — Evaluation of TFG aggregates in HMSN-P patient spinal MNs. (A) Immunostaining for TFG in control and HMSN-P patient SMI-32-positive neurons. Scale bar = 10 μm. (B) Quantification of area of TFG-positive puncta in SMI-32-positive neurons measured by high-content analysis (n = 3, n.s. by Student t-test). Error bars are ± s.e.m. (C) Immunostaining for TFG in purified MNs by HB9::GFP sorting plated on monolayer of primary cortical mouse glia. Scale bar = 10 μm. (D) Quantification of area of TFG-positive puncta in purified MNs measured by high-content analysis (n = 3, n.s. by Student t-test). Error bars are ± s.e.m. TFG aggregates were not significantly increased in purified MNs when they were co-cultured with mouse glia. (E) Immunocytochemical analysis with anti-multi-ubiquitin chain antibody (FK2) using control and HMSN-P patient iPS-MNs. Aggregations of multi-ubiquitin were not detected in HMSN-P patient iPS-MNs. (PDF 509 kb) [file 13041_2017_286_MOESM2_ESM.pdf]

Figure S3

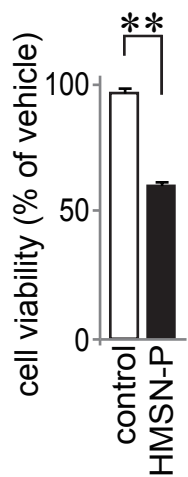

Supplement: Additional file 3: Figure S3. — Vulnerability of HMSN-P patient iPS-MNs against UPS inhibitory stress. Survival of control and HMSN-P patient iPS-MNs after bortezomib exposure (n = 3, **p < 0.01, by Student t-test). (PDF 222 kb) [file 13041_2017_286_MOESM3_ESM.pdf]
