# Supplementary material for: Proteasome impairment in neural cells derived from HMSN-P patient iPSCs
Source: Mol Brain. 2017 Feb 15;10:7. doi: 10.1186/s13041-017-0286-y (PMC5310050; doi:10.1186/s13041-017-0286-y)
Supplement: Additional file 4: Table S1. — Primers used for editing TFG gene. (DOCX 12 kb) [file 13041_2017_286_MOESM4_ESM.docx]

**Table S1:** Primers used for editing *TFG* gene

| primer | sequences |
| --- | --- |
| primer A | 5'- TATCTTTGTATTTCTGATCCTAC -3' |
| primer B | 5'- GCGACGGATTCGCGCTATTTAGAAAG -3' |
| primer C | 5'- CGTCAATTTTACGCATGATTATCTTTAAC -3' |
| primer D | 5'- TAATGGATTCAATCATCTTTATTAAC -3' |
